# Pre-service healthcare professionals attitudes and self-efficacy towards individuals with intellectual disability in Ghana

**DOI:** 10.1186/s12909-023-04697-0

**Published:** 2023-09-28

**Authors:** Maxwell Peprah Opoku, Hala Elhoweris, Michael Amponteng, William Nketsia, Eric Lawer Torgbenu, Reuben Saah

**Affiliations:** 1 Special Education Department, United Arab Emirates University, P. O. Box 15551, Al-Ain, United Arab Emirates; 2 School of Education, Western Sydney University, Sydney, Australia; 3 Department of Physiotherapy and Rehabilitation Sciences, University of Health and Allied Sciences, Ho, Ghana; 4 University of Technology Sydney, Faculty of Health, Sydney, Australia; 5 Abetifi Presbyterian College of Education, Library Section, Abetifi, Ghana

**Keywords:** Healthcare students, Intellectual disability, Training, Discrimination, Subjugation, Ghana

## Abstract

**Background:**

Intellectual disability (ID) involves impairment of general mental abilities, restricting the participation of individuals in conceptual, social and practical activities. Consequently, rehabilitation services are critical in efforts towards promoting the social and educational inclusion of persons with ID. However, the preparedness of health professionals in performing such a role depends on their perceptions of individuals with ID. Ajzen’s theory of planned behaviour was used as theoretical framework to understand the relationship between the perceived attitude and self-efficacy of healthcare students towards persons with ID.

**Method:**

A cross-sectional survey was conducted among healthcare students (*N* = 328) in a Ghanaian university. The Community Living Attitude Scale for ID(CLAS-ID) and General Self-efficacy (GSE) Scale were employed to assess their attitudes and self-efficacy towards people with ID respectively. The data were analysed using SPSS and AMOS and were subjected to a t-test, ANOVA, correlation and regression.

**Result:**

The healthcare students were ambivalent about both self-efficacy and attitudes towards persons with ID, and there was correlation between attitudes and self-efficacy. Attitudes and self-efficacy also varied across the demographic characteristics of the respondents including age, having a relative with ID, level of study, religion, and programme of study.

**Conclusion:**

The study underscores the necessity for healthcare curriculum reform and provides corresponding recommendations. The study emphasizes the importance of enhancing healthcare students' understanding of ID, changing their attitudes, and bolstering their self-efficacy. This is crucial to foster positive attitudes, confidence in providing support to individuals with ID, and raising awareness within the broader community. To achieve this, health educators are encouraged to incorporate exposure to individuals with ID into healthcare students' training, along with more structured field experiences designed to increase their contact and interaction with individuals with ID. Such initiatives would enable students to better understand the uniqueness and requirements of individuals with ID.

## Introduction

Rehabilitation is the practice of designing programmes or policies aimed at reducing the effects of impairment on individuals [1]. Most importantly, rehabilitation is aimed at enabling persons with disabilities to function and maximise their potential in society [2]. This could be in the form of developing programmes to enhance the participation of persons with disabilities in all aspects of society such as health, education, employment and transportation [1–3]. However, in most sub-Saharan African countries, such as Ghana, there are limited interventions for rehabilitating persons with disabilities, which have been linked to poor understanding, negative attitudes and limited intervention programmes [4, 5]. In an effort towards promoting the well-being of persons with disabilities, health workers are important stakeholders who are expected to be the change agents as well as create awareness among the larger population. This means that future health workers ought to be prepared and provided with the requisite knowledge, skills and approaches to promote the well-being of persons with disabilities [6]. Indeed, the most marginalised disability group in most developing countries are individuals with intellectual disability (ID). Unfortunately, there is scant literature on the attitudes of future health workers towards persons with ID in sub-Saharan African countries, including Ghana.

The behavioural challenges associated with ID cannot be overemphasised. ID is characterised by significant deficits in both intellectual functioning and adaptive behaviour, including many social and practical skills [7]. The onset of ID is usually diagnosed during the developmental period (before 18 years), with leading causes mostly attributed to genetic factors and neurodevelopmental, neurological and other bio-healthcare conditions. In developing countries, including sub-Saharan African countries such as Ghana, approximately 10 to 15 individuals per every 1,000 persons are believed to be living with a form of ID, while between one and three percent of the population in Western countries are living with a form of ID [8]. Empirical evidence from the perspectives of parents and people with ID suggests that people with ID continue to suffer various forms of discrimination and rejection in sub-Saharan African countries [4, 9]. This underscores the need for health workers to help educate society about the aetiology of ID as well as the acceptance and support services required by persons with ID.

In Ghana, there is a strong traditional understanding of ID [9, 10] as being linked to superstition. Specifically, the onset of ID has consistently been linked to supernatural forces believed to have visited the Earth [9]. In some instances, they are perceived as ‘children of rivers’ who have visited families for past bad deeds. Based on these perceptions, people with ID are usually labelled ‘*nsuoba*’, translated as ‘*children of river*’, idiots, imbeciles and ‘things’ [11, 12]. These perceptions are so prevalent that families are stigmatised if any member is born with ID [9]. Moreover, it is believed that ID is transferable, and thus, people do not want to be associated with anyone suspected of having ID. The presence of children with ID in families becomes a burden on families as they have to deal with negative perceptions and limited rehabilitation centres [9, 12]. While there is legislation promoting the well-being of persons with ID, such as the Persons with Disability Act 715 [13], persons with ID continue to suffer or be subjugated in Ghanaian society [10]. In order to re-write the story of persons with ID, there is a need for a campaign led by health workers to raise awareness about the capabilities of persons with ID as well as the causes of ID.

Despite the limited nature of studies about healthcare students’ attitudes towards persons with ID in Ghana, studies elsewhere have provided a framework within which to situate the current study. Some studies have attempted to understand the attitudes of students towards individuals with ID [14–19]. In a cross-national study of Libya and the UK, Benomir and colleagues [19] found attitudinal differences between the participants in their study. While the Libyan students were less positive, those in the UK were more positive towards individuals with ID. Similar studies were conducted in the UK to compare the attitudes of White British and British Asians towards individuals with ID [17]. Once again, there were differences between the participants based on their ethnicity. While British Asians were less positive about exclusion and similarity, White British people were more positive about such measures. Importantly, all the above studies were conducted on general cohorts of students and did not limit the study samples to a core group, such as healthcare students.

The fluidity regarding attitudes towards persons with ID has been noted in the literature [20, 21]. In particular, a range of variables have been found to influence attitudes towards persons with ID. For example, in a Pakistani study conducted by [22] on attitudes towards persons with ID, religion and gender were shown to be associated with communal attitudes. Specifically, females were found to have more favourable attitudes towards persons with ID compared to males. However, studies conducted in other contexts found no relationship between gender [19, 23] and religion [17] and attitudes towards persons with ID. Other inconsistencies have also been reported in relation to age, contact with persons with ID, relatives with ID and the influence on attitudes towards persons with ID [20, 21, 24–26].

With limited studies on attitude towards persons with ID in sub-Saharan Africa, it is important to develop a contextual understanding of the variables influencing attitudes towards persons with ID. Most importantly, evidence suggests that some healthcare professionals lack the requisite skills and knowledge when it comes to supporting individuals with disabilities [27]. To provide effective care for individuals with disabilities, especially those with ID, healthcare students must possess positive attitudes and the requisite self-efficacy [28] towards service provision to individuals with ID. Healthcare professionals are expected to play crucial roles in providing services to individuals with ID, including the tasks of raising awareness and educating the broader society about ID. Consequently, conducting such a study will offer evidence that may reveal deficiencies in healthcare students’ attitudes and self-efficacy related to disability issues, thus informing their training requirements in this domain.

### Theoretical framework

Intention mirrors actual behaviour which has contributed to the use of theory of planned behaviour (TPB) to guide studies. In this study, Ajzen TPB [28] which has been widely used to understand people’s intentions towards a given behaviour [29]. The theory was an extension of the theory of reasoned behaviour, which suggested that behaviour was a product of two related variables: normative beliefs and behavioural beliefs [30]. Normative beliefs refer to the influence of social pressure on individuals in the performance of a behaviour, while behavioural beliefs refer to one’s perception towards a behaviour. However, Ajzen [28] identified a third variable – control belief – as a potential influence on individual attitudes towards a behaviour. Ajzen [29] proposed that a third of related beliefs influence people’s intention towards a given behaviour. Control beliefs refer to a person’s confidence in their ability to perform a given behaviour. According to Ajzen [28], a positive interaction among these three related beliefs leads to favourable intentions towards a behaviour.

In this study, the components of TPB were conceptualised as follows: Behavioural beliefs develop into attitudes towards persons with ID, that is, the perception of students towards persons diagnosed with ID. Normative beliefs develop into subjective norms, that is, the influence of social pressure on the intention of students towards persons with ID. Furthermore, control beliefs accumulate into self-efficacy, thereby explaining the influence of a person’s confidence on their intention towards persons with ID. While these variables are important, according to Ajzen [28, 29], background variables could serve as additional explanations behind intention. This suggests that in order to develop an in-depth understanding of a given phenomenon, consideration ought to be given to the background variables that can impact the related beliefs. However, the study reported here focused on attitudes and self-efficacy, which are directly linked to individuals, such as healthcare students. Unfortunately, to the best of the best of our knowledge, the relationship between attitudes and self-efficacy towards persons with ID is understudied. To address this gap in the literature, this study was guided by the following research questions:

- What is the association among healthcare students’ profile, attitudes and self-efficacy towards persons with ID in Ghana?
- What is the relationship between healthcare students’ self-efficacy and attitudes towards persons with ID in Ghana?
- Do healthcare students’ self-efficacy predict their attitudes towards persons with ID in Ghana?

## Methods

### Study participants

The study participants were healthcare students enrolled at public university in Ghana. Since health workers are central in efforts towards promoting the social inclusion of persons with ID, we conveniently recruited the study participants from a university established to train health workers. The inclusion criteria were as follows: a) must be enrolled in the selected institution; b) at various stages of undergraduate study; c) have a fair knowledge or understanding of ID and c) at least 18 years and able to read and sign the consent form.

The study sample was estimated using *OpenEpvi version 3* which is an open source sample size calculator (http://www.openepi.com/SampleSize/SSCC.htm). It has been estimated that 10—15 per 1000 of the population is living with a form of ID in Africa [8]. With this mind, parameters for the sample size calculation were set as follows:

- Confidence Interval = 99%
- Hypothesized % frequency of outcome factor in the population = 50%
- Confidence limit = 15%
- Design effect = 1
- Confidence interval = 99.99%
- (http://www.openepi.com/SampleSize/SSPropor.htm)

The computation yielded expected sample size of 169 for this study. However, 328 data were collected from pre-service students and thus, exceeding our target. Out of the 400 questionnaires, 328 were return suggesting a response rate of 82%.

Table 1 summarises the demographic information of the 328 healthcare students recruited for this study. Fifty-four percent (54%) of the participants were male compared to 46% female. In terms of age, 55% were between the ages of 18 and 21 years compared to 45% who were at least 22 years. Furthermore, 79% indicated that they were enrolled in allied health courses such as physiotherapy, healthcare laboratory and public health, while 15% were trainee doctors. While 80% said that they did not have a relative with ID, 20% indicated otherwise. On religion, 68% were Christian, while 9% were Muslim (see Table 1 for further details).

**Table 1:** Association between students’ profile, attitude and self-efficacy

Category (*N* = 328)	Sample (%)	Self-Efficacy	Attitudes	Exclusion	Similarity	Empowerment	Sheltering
***Gender***							
Male	177 (54%)	3.73 (.96)	3.16 (0.66)	2.29 (1.03)	4.33 (1.07)	3.40 (1.22)	3.63 (0.99)
Female	151 (46%)	3.65 (.70)	3.20 (0.67)	2.25 (1.02)	4.57 (1.31)	3.48 (1.07)	3.64 (1.05)
*t*		.87	-.56	.38	-1.55	-.59^#^	-.15
*Partial eta squared*		.002	.001	.001	.007	.001	.001
***Age***							
18–21	181 (55%)	3.83 (0.60)	3.26 (0.70)	2.13 (1.06)	4.83 (1.27)	3.64 (1.19)	3.78 (1.10)
≥ 22	147 (45%)	3.53 (1.01)	3.08 (0.61)	2.44 (0.96)	3.96 (1.36)	3.19 (1.06)	3.46 (0.87)
*t*		3.08**	2.45*	-2.75**	6.00**	3.57**	2.91**
*Partial eta squared*		0.03	.02	.02	.10	.04	.02
***Relative with ID***							
Yes	64 (20%)	3.27 (0.76)	3.17 (0.54)	2.56 (0.84)	3.99 (1.34)	3.22 (1.07)	3.58 (0.81)
No	264 (80%)	3.80 (0.84)	3.18 (0.69)	2.20 (1.06)	4.55 (1.37)	3.48 (1.17)	3.65 (1.06)
*t*		-4.59**	-.15	2.50**	-2.92**	-1.62	-.59#
*Partial eta squared*		.06	.001	.02	.03	.008	.001
***Level of Study***							
Year 1	42 (13%)	3.75 (0.66)^a,b,c^	2.96 (0.61)^a^	1.68 (0.68)^a^	4.83 (1.69)^a^	3.35 (1.36)^a^	3.51 (1.14)^a^
Year 2	76 (23%)	3.95 (0.43)^a,,b^	3.35 (0.73)^b^	2.27 (1.12)^b^	4.84 (1.04)^a^	3.59 (1.29)^a^	3.94 (1.10)^a,b^
Year 3	168 (51%)	3.45 (1.01)^a,c^	3.14 (0.65)_a,b_	2.49 (0.98)^b,c^	4.05 (1.35)^b^	3.29 (1.04)^a,b^	3.46 (0.95)^a,c^
Year 4	42 (13%)	4.17 (4.43)^a,b,c^	3.25 (0.62)^a,b^	1.96 (0.54)^a,b^	4.90 (1.27)^a^	3.81 (1.03)^a,c^	3.87 (0.80)^a^
*F*		17.13**	3.68##**	14.04##**	10.93##**	2.96*	5.09##**
*Partial eta squared*		.11	.03	.08	.09	.03	.05
***Programme***							
Trainee Doctors	48 (15%)	3.78 (0.60)	3.00 (0.61)	1.63 (0.54)^a,b^	5.03 (1.43)^a^	3.53 (1.43)	3.48 (1.11)
Nurses/midwives	20 (6%)	3.70 (0.65)	3.23 (0.44)	2.03 (0.96)^a,b^	5.13 (0.70)^a^	3.13 (1.15)	3.98 (0.82)
Allied Professionals	260 (79%)	3.68 (0.90)	3.21 (0.69)	2.41 (1.05)^a,c^	4.28 (1.38)^c^	3.44 (1.10)	3.63 (1.01)
*F*		0.27	2.10	29.37##**	14.51##**	0.78	1.70
*Partial eta squared*		.002	.01	.08	.05	.005	.01
***Religion***							
Christian	224 (68%)	3.71 (0.90)^a,b,c^	3.15 (0.63)^a,b,c^	2.23 (0.99)	4.44(1.32)^a,b,c^	3.40 (1.11)^a^	3.61 (1.02)^a,b,c^
Muslim	30 (9%)	3.33 (0.81)^a,b^	2.91 (0.72)^a,b^	2.41 (0.90)	3.83 (1.56)^a,b^	2.71 (1.19)^b^	3.25 (0.93)^a,b^
Traditionalists	74 (23%)	3.81 (0.62)^a,c^	3.37 (0.72)^a,c^	2.34 (1.18)	4.69 (1.42)^a,c^	3.82 (1.13)^c^	3.86 (0.99)^a.c^
*F*		3.55*	5.88**	0.70##	4.20*	10.69**	4.18*
*Partial eta squared*		.02	.04	.004	.03	.06	.03
***Contact with ID***							
Frequently	81 (25%)	3.76 (1.19)	3.29 (0.68)	2.45 (1.09)	4.38 (1.41)	3.54 (1.26)	3.75 (0.95)
Occasionally	77 (23%)	3.65 (0.76)	3.10 (0.65)	2.12 (0.96)	4.39 (1.36)	3.42 (1.08)	3.62 (1.03)
Rarely	170 (52%)	3.69 (0.85)	3.16 (0.67)	2.25 (1.02)	4.49 (1.38)	3.39 (1.14)	3.57 (1.04)
*F*		0.24	1.76	2.11	0.24	0.46	1.04
*Partial eta squared*		.002	.01	.01	.001	.003	.006

Superscripts ^(a,b,c)^ means significant difference between groups

*ID* Intellectual Disability, *#* Violation of homogeneity of variance with results of within groups reported, *##* Violation of homogeneity of variance with results of Welch statistics reported

^*^p < .05

^**^p < .01

### Instrument

The questionnaire used for the data collection consisted of three parts. The first part collected information on the demographic characteristics of the participants, such as gender, age, year of study, whether they had a relative with ID, frequency of contact with persons with ID and religion. The researchers’ decision regarding which demographic variables to include in this study was based on a review of the literature (e.g. [20, 22, 23]).

Part two of the questionnaire was the Community Living Attitude Scale for ID (CLAS-ID) [31]. The original scale was validated in the sub-Saharan African context prior to being used for this study (In press). The scale consisted of 17 items rated on a five-point Likert-type response format ranging from 1 (strongly disagree) to 5 (strongly agree). The scale comprised four main factors: empowerment, exclusion, sheltering and similarity. While empowerment refers to the self-belief and capacity of persons with ID to take ownership and make decisions concerning their lives, exclusion refers to the extent to which people think that individuals with ID should be excluded from others. Also, while sheltering refers to the help needed by individuals with ID to keep safe, similarity refers to the extent of similarity between persons with ID and others. The scale was sent to five university lecturers in special education to examine its content and appropriateness for data collection. Three of the academics responded in writing that the content was valid; however, the remaining two suggested iterations, which were incorporated into the draft used for the data collection.

Some example items were as follows: ‘A person would be foolish to marry a person with intellectual disability’, ‘People with intellectual disability should live in special facilities because of the dangers of life in the community’ and ‘People with intellectual disability can be productive members of society’.

The third part of the questionnaire was the revised General Self-efficacy (GSE) Scale, which was adapted from Schwarzer and Jerusalem [32] and was originally designed to measure an individual’s general confidence. However, we adapted it by adding phrases to measure the perceived efficacy of students towards individuals with ID. This scale consists of 10 items rated on a five-point Likert-type format ranging from 1 (not at all true) to 5 (exactly true). The scale was subjected to content validation by academics and pre-testing before it was used for data collection. Some of the items on the revised scale were as follows: ‘I can always manage to solve difficult problems faced by individuals with ID’, ‘If an individual with ID hurts or assaults me, I can find the means and ways to get what I want him or her to do’, and ‘It will be easy for me to stick to my aims and accomplish my goals of managing the behaviour of individuals with ID.’

The reliability of the scales was checked using Cronbach’s alpha. The results showed that the scales yielded a suitable score: the GSE = 0.71, the CLAS-ID = 0.08, and the sub-scales (exclusion = 0.90, similarity = 0.88, empowerment = 0.70 and sheltering = 0.60). The relevance of reporting the mean scores (sum scores divided by the number of items) in the present study was to facilitate the understanding and interpretation of the results. Thus, a mean score of at least 4 represented a positive attitude towards persons with ID.

### Procedure

Ethics approval was received from the University of Health and Allied Sciences prior to the data collection. Subsequently, letters were sent to the various departments in the school, seeking permission to collect data from the students. The data were collected by the fourth and fifth authors. The authors visited lecture halls and waited for the students until the end of their classes before distributing the questionnaires to those who met the inclusion criteria and consented to participating in the study. The students were informed of the purpose of the study and the inclusion criteria for participation before the questionnaire was distributed. They were told about the voluntary nature of their participation and that there would be no financial reward, incentive or other material gain for the completion of the questionnaire. They were also made aware of confidentiality and that their identity, department or class would not be included in the reporting of the study.

Within one week, the authors attended the same classes to retrieve the completed questionnaires directly from the students. Each student spent approximately 20 to 25 min on the questionnaire, which was written in English. All the participants signed an informed consent before taking part in this study. The period for the data collection was between March 2018 and November 2019.

### Data analysis

The data were analysed using SPSS, version 26, and AMOS. After the data collection, the fifth author entered the data in Excel before transferring them to SPSS for analysis. Preliminary analyses were made to ensure that the data were appropriate for the parametric test. Observations of the histograms showed that the data were normally distributed [33]. At this stage, the mean scores of the CLAS-ID and GSE were checked before proceeding to answer the research questions.

To answer research question 1, t-tests and analyses of variance (ANOVA) were computed to assess the association between the demographic profiles of the students and the CLAS-ID and GSE. While t-tests were computed for the two-level demographic profiles (e.g. gender and age), ANOVAs were computed for demographic variables with at least three levels (e.g. contact with persons with ID and level of study). Here, an analysis of homogeneity of variance was assessed using the results of Levene’s test. With respect to the t-test, the within-groups results were reported. With respect to the ANOVA, the results of the Welch statistics were reported when the assumption of homogeneity of variance was violated. The magnitude of the effect of the t-tests and ANOVAs was assessed using partial eta squared as follows: small = 0.01 to 0.05; moderate = 0.06 to 0.9; and large = at least 1 [34].

To answer research question 2, AMOS was used to assess the causal relationship between self-efficacy and attitudes. The following fit indices were used to determine appropriateness of the model: chi-square of below 5, comparative fit index (CFI) and Tucker-Lewis Index (TLI) of at least 0.09 and RMSEA and SRMR of below 0.08. Also, each item was expected to yield a regression weight of at least 0.50 for retention in the study [35–37]. Here, the correlations were interpreted as follows: small (*r* = 0.10 to 0.29); medium (*r* = 0.30 to 0.49); and large (*r* = 0.50 to 1.0) [34].

To answer research question 3, hierarchical regressions were computed to ascertain the predictors of attitudes. Preliminary checks were conducted to ensure that the following assumptions were not violated: normality, homoscedasticity and linearity [34]. In step 1, self-efficacy was regressed directly on attitudes. In step 2, the demographic variables were added to the model.

## Results

The overall mean attitude of the healthcare students was 3.18 (SD = 0.67), and the means of the sub-scales were as follows: exclusion (M = 2.27; SD = 1.03), similarity (M = 4.44; SD = 1.38), empowerment (M = 3.43; SD = 1.15) and sheltering (M = 3.63; SD = 1.01). Also, the mean score on the GSE was 3.70 (SD = 0.85).

### Association among demographics, attitudes and self-efficacy

An independent samples t-test was computed to assess the association between the two-level demographics, attitudes and self-efficacy (Table 1). With respect to the t-tests, differences between participants were found for only two demographics: age and having a relative with ID. To elaborate, there were age-related differences between the participants on all the measures. For the GSE, the younger participants were more efficacious than those who were at least 22 years old *t* (326) = 3.08, *p* = 0.002. However, the effect size was very small. Also, for the CLAS-ID, the younger healthcare students were more positive than those who were older, t (326) = 2.45, *p* = 0.02, with a small effect size. Similar trends were observed on all the sub-scales, with the exception of exclusion, where older students were more in favour of the exclusion of persons with ID than their younger counterparts.

In addition, significant differences were observed regarding having a relative with ID, self-efficacy and attitudes. In terms of self-efficacy, the participants who indicated that they did not have a relative with ID were more efficacious than those who indicated otherwise, t (326) = -4.87, *p* = 0.001. Although there was no difference between the participants on the CLAS-ID, interesting observations were made on the sub-scales. While those who had a relative with ID were more in favour of more exclusion *t* (326) = 2.50, *p* = 0.01, those without a relative with ID indicated more similarities between persons with ID and other members of society, *t* (326) = -2.92, *p* = 0.004. Once again, the effect sizes were very small.

A one-way ANOVA was computed to assess the relationship between the three-level demographics, attitudes and self-efficacy of the healthcare students towards persons with ID (see Table 1). Significant differences were observed among three demographic factors: level of study, religion and programme of study. First, differences were observed between the participants on overall attitudes, *F* (3, 324) = 3.68, *p* = 0.01, with a small effect size (partial eta squared = 0.03).

Post-hoc comparisons using the Tukey HSD test indicated that healthcare students in their second year were more positive than those in their first year. However, no difference was found between those in their third and fourth years. In terms of the GSE, a significant difference was observed between the participants, *F* (3, 324) = 12.63, *p* = 0.001. A post-hoc comparison using the Tukey HSD test found differences between the participants in their third and fourth years only.

Also, differences were observed between the participants in the area of religion. For the CLAS-ID, those healthcare students who identified as traditionalists were more positive than those who identified as Muslim and Christian, *F* (2, 325) = 5.88, *p* = 0.003. However, the effect size was very small. A post-hoc comparison using the Tukey HSD test found significant differences between Muslims and traditionalists. Similar observations were made on almost all the sub-scales, with the exception of empowerment where there was significant difference between all the participants. Specifically, participants who identified as traditionalist (a belief in the African religious system) were more in favour of empowerment than those who identified as Christian and Muslim. For the GSE, those who identified as traditionalist recorded higher self-confidence than others, *F* (2, 325) = 3.55, *p* = 0.03, with a small effect size. A post-hoc comparison using the Tukey HSD test showed significant differences between the participants.

Although there were no differences between the participants regarding the CLAS-ID, differences were observed for two sub-scales. With respect to exclusion, participants who were allied health professionals were more positive on attitudes than those who were enrolled in healthcare and nursing/midwifery programmes, F (2, 325) = 13.32, *p* = 0.001. A post-hoc comparison using the Tukey HSD test showed significant differences between healthcare students enrolled in allied health courses and healthcare doctors. However, neither group differed from those enrolled in nursing/midwifery programmes. Additionally, on similarity, those enrolled in nursing/midwifery programme were more positive than those enrolled as trainee doctors and in allied health programmes. A post-hoc comparison using the Tukey HSD test found significant differences between the participants enrolled in allied health programmes and others.

### Relationship between self-efficacy and attitude

The relationship between self-efficacy and attitude towards individuals with ID were measured using Pearson’s correlation coefficient. With respect to the causal relationship between attitude and self-efficacy, the bi-directional arrow showed a moderate (*r* = 0.58) causal relationship between the two latent variables (see figure below for details).

**Fig. 1:**
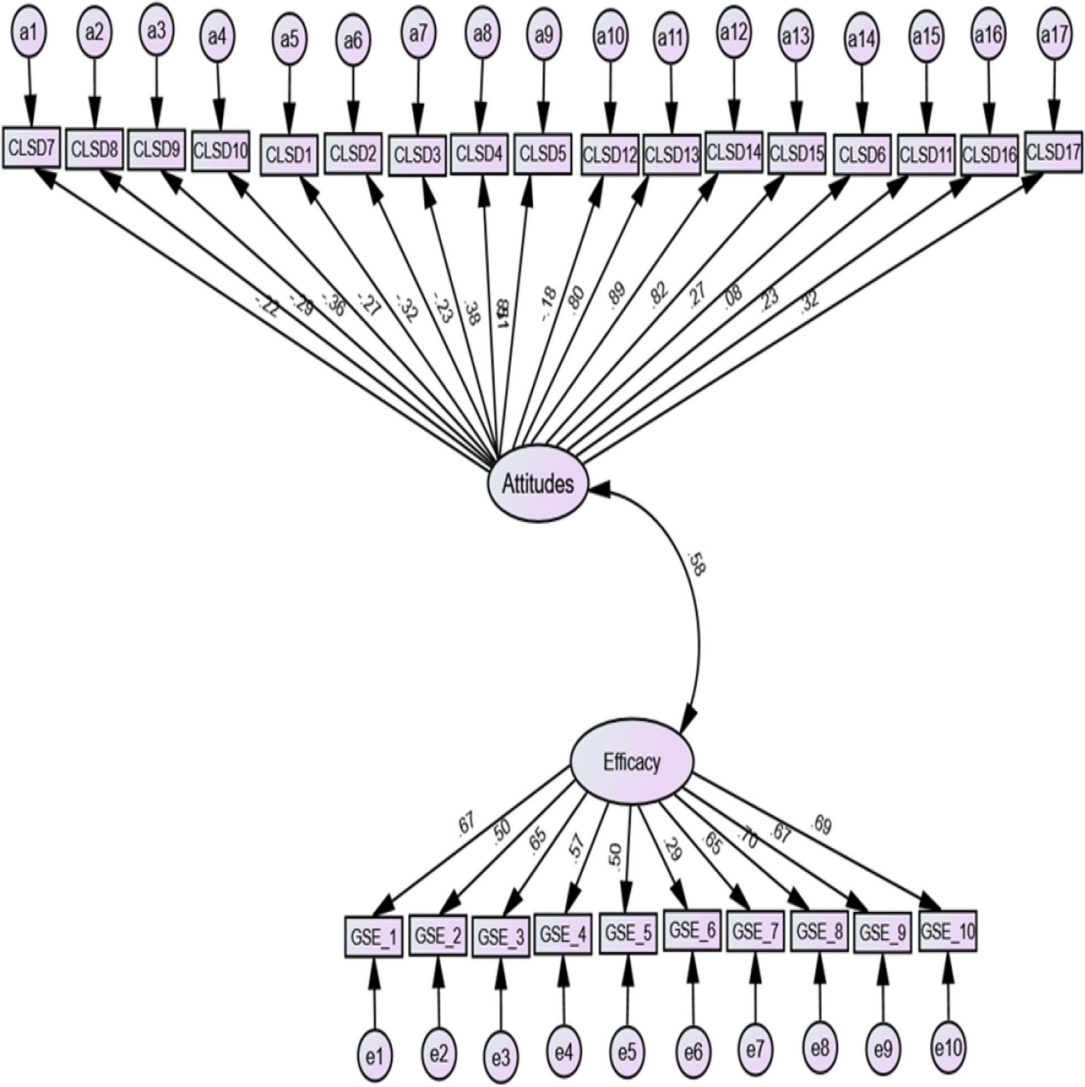
Association between attitudes and self-efficacy

Figure 1 presents the causal relationship between the attitude sub-scales and self-efficacy. However, the model was deemed appropriate as it yielded the following fit indices: chi-square = 4.36, TLI = 0.91, CFI = 0.93, RMSEA = 0.07 and SRMR = 0.05. While very small causal relationships were observed between self-efficacy, exclusion (*r* = -0.29) and empowerment (*r* = -0.28), there were moderate relationships between self-efficacy and similarity (*r* = -0.55) and self-efficacy and shelter (*r* = 0.32).

**Fig. 2:**
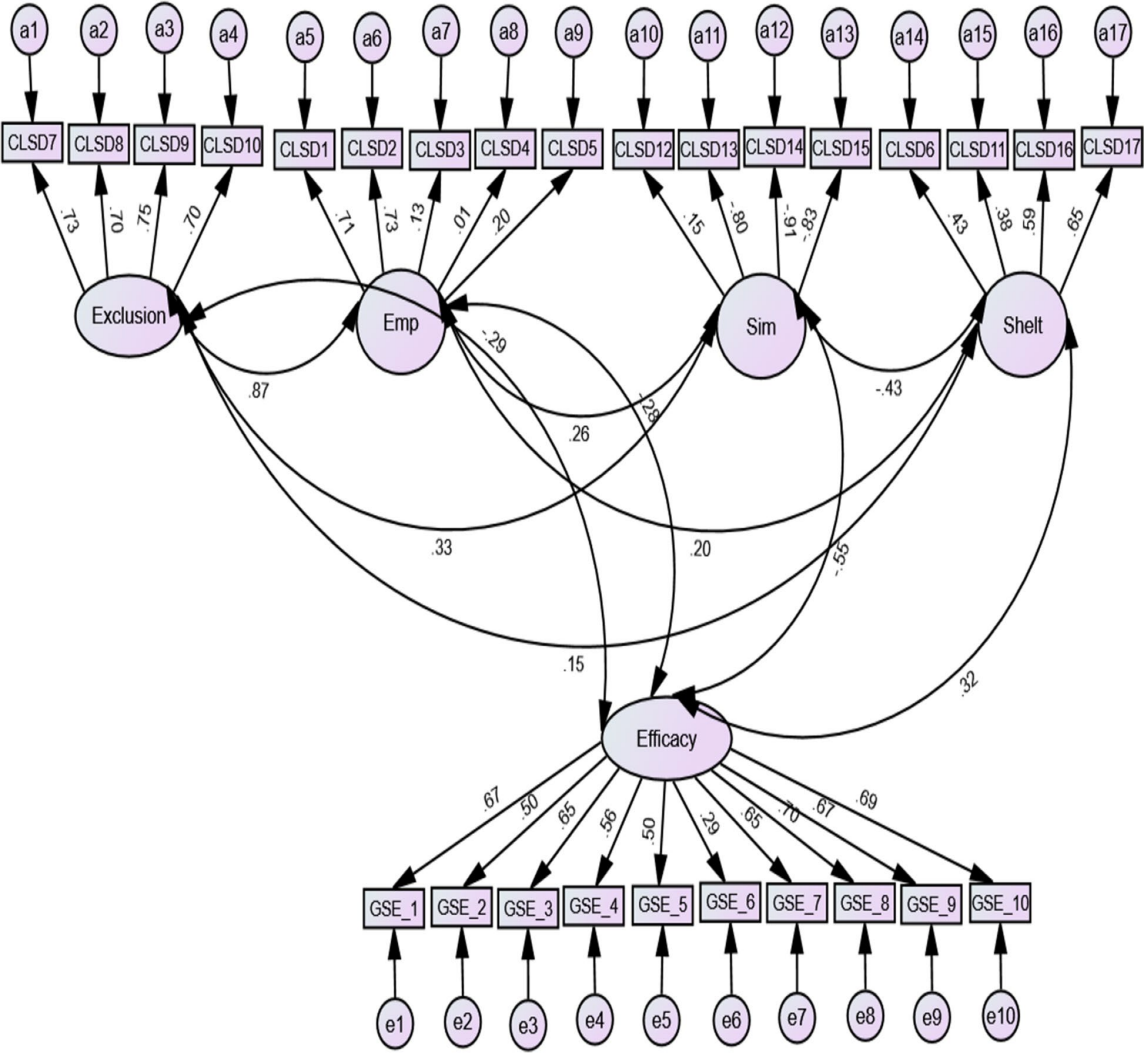
Relationships between the sub-scales and self-efficacy

Other observations were made between the sub-scales (see Fig. 2). Once again, the fit indices were as follows: chi-square = 4.36, TLI = 0.91, CFI = 0.93, RMSEA = 0.07 and SRMR = 0.05. For example, there was a large correlation between exclusion and empowerment (*r* = 0.87) and moderate correlations between similarity and shelter (*r* = -0.430) and exclusion and similarity (*r* = 0.33).

### Predictors of attitudes

A hierarchical regression was used to assess the importance of self-efficacy in developing healthcare students’ attitudes towards persons with ID in Ghana (see Table 2). In step 1, self-efficacy was regressed directly on attitudes. The results showed that self-efficacy (*beta* = 0.16, *p* = 0.01) made a significant contribution, accounting for 21% of the variance in attitudes, *F* (1, 326) = 8.17, *p* = 0.005.

**Table 2:** Summary of predictors of attitudes

Category	*B*	*SE B*	*β*	*t*	*p*
***Step 1***					
Self-efficacy	.21	.07	.16	2.86	.01**
***Step 2***					
Self-efficacy	.19	.08	.14	2.54	.01**
Gender	.56	1.29	.02	0.43	.67
Age	-3.52	1.40	-.16	-2.52	.01**
Religion	1.42	0.76	.11	1.87	.06
Level of study	1.41	0.80	.11	1.76	.08
Programme of study	1.58	.86	.10	1.84	.07
Relative with ID	-1.40	1.62	-.05	-.86	.39
Contact with Persons with ID	-.98	.76	-.07	-1.28	.20

*ID *Intellectual Disability

^*^p < .05

^**^p < .01

In step 2, the addition of the demographic variables contributed to a marginal increase of the variance in attitudes by 0.5%, increasing the total to 21.5%. The overall model contributed significantly to the variance in attitudes, *F* (8, 319) = 3.27, *p* = 0.001. In step 2, though self-efficacy (*beta* = 0.14, *p* = 0.01) made a significant contribution to the variance in attitudes, age (*beta* = -0.16, *p* = 0.01) made the most significant contribution to the variance in attitude.

## Discussion

This study aimed to assess the attitudes and self-efficacy of healthcare students towards individuals with ID in Ghana. It was conducted against the backdrop of the central role of health workers providing services to individuals with ID including raising awareness and educating the general society about ID. The study found positive interactions between self-efficacy and attitudes towards individuals with ID. As the self-efficacy of the students increased, attitudes improved. Furthermore, the importance of self-efficacy in attitudes was confirmed by the results of the regression. The findings of this study provide support Ajzen [28] for the proposition regarding the positive interaction between attitudes and self-efficacy towards a given phenomenon. It appears that changing the attitudes of health workers may not be sufficient on its own as other affective variables have to be developed to propel future health workers into being change agents. In the Ghanaian context, other variables such as improving the self-efficacy of healthcare students may be needed as part of efforts towards teaching future students to understand the aetiology of ID and supporting individuals with ID. There is the possibility for future health workers to be exposed to curricular on ID; however, such programmes need to be augmented alongside improvements in self-efficacy.

According to Ajzen [28], attitude is critical to promoting the social acceptance of a given behaviour. In this study, the mean scores showed the ambivalence of healthcare students towards individuals with ID, suggesting that the healthcare student participants were unsure about their attitude towards individuals with ID. This finding is slightly inconsistent with the results of previous studies which reported either negative or positive attitudes towards persons with ID [22, 23, 26]. In Ghana, the understanding of ID is guided by traditional beliefs about disability. Here, the onset of ID is poorly understood, which contributes to the discrimination and subjugation of people with ID [9, 12, 33, 38]. There is the possibility for healthcare students to be exposed to courses on disability. However, the participants found themselves in an environment where ID has negatively intersected with the local tradition. The study participants shared a similar culture with the larger society. As such, they might have heard or shared in the societal perceptions of persons with ID. While the students may have been enlightened about ID, it appears that they have been influenced by the local culture and practice, suggesting that more work needs to be done to overcome the inherent negativity towards individuals with ID.

Self-efficacy was Ajzen’s [29] main contribution in the conception of TPB. According to Ajzen [29], self-efficacy can have a direct influence on behaviour. In this study, the mean score indicated the neutrality of the participants regarding their self-efficacy towards individuals with ID. In order to promote a given behaviour, there is a need for high self-efficacy before the behaviour can even take place. However, the participants’ level of neutrality suggests that they might not have been confident in the ability to provide support services to persons with ID and educate society about their capability and acceptance as equal members of society. Indeed, many previous studies have reported on the inability of health workers to provide effective services to persons with ID [39, 40]. The lack of understanding of ID appears to have influenced the poor health service provision to persons with ID [41]. It is possible that developing the confidence of healthcare students would put them in a better position to identify and provide the services required by individuals with ID. There is also the possibility that healthcare students will be armed with the requisite knowledge and skills to enable them confidently engage or respond to ‘deficit talk’ about ID. Arguably, healthcare students in Ghana cannot provide effective services and public education about ID so long as they are ambivalent about self-efficacy.

In seeking to provide additional insight into attitudes and self-efficacy, Ajzen [28] addressed the importance of background variables. This study found no relationship between the participants on contact with persons with ID. This is a surprising finding because several studies have reported on the positive interactions between attitudes and contact with individuals with ID [26, 42]. Nevertheless, in this study, this finding could be linked to the quality of interaction between the healthcare students and individuals with ID. Previous studies have suggested that the more students interact with persons with ID, the more likely there would be changes in their perception [40, 43]. This suggests that in the Ghanaian context, the training of health workers may not be based on allowing students to spend time with individuals with ID in order to develop an understanding of their capabilities. Thus, the training can be described as more theoretical in nature as there is no deliberate attempt at enabling students to come in contact with individuals with ID. It is understandable that the lack of difference between the participants was based on the nature of their contact with persons with ID. As contact with persons with ID is fundamental to changing attitudes, health educators could consider including healthcare students’ exposure to individuals with ID as part of their training.

An interesting observation was made regarding having a relative with ID. While those with a relative with ID were more in favour of the exclusion of persons with ID, those without a relative with ID indicated that persons with ID were more similar to other people in society. Also, those without a relative with ID were more efficacious than those with a relative with ID. This finding arguably shows that the healthcare students’ relationship with their relatives with ID has not influenced their self-efficacy and attitudes. In Ghana, the negativity surrounding persons with ID cannot be overstated [9, 12]. Individuals with ID are believed to be the most excluded and stigmatised among the disability population [38]. Regardless of the calibre of the person, they tend not to be associated with persons with ID. This appears to have been the case for the participants with a relative with ID, who were more in favour of more exclusion of persons with ID. In training healthcare students, health educators should be able to target those with relatives with ID and encourage them to accept and support their relatives with ID. Once they have reached the point of accepting their family member with ID, they will be in a better position to educate others about positive behaviour support and support services available for individuals with ID.

This study also noted the association between religion and attitudes towards individuals with ID. Specifically, participants who identified as traditionalist appeared to be more positive about attitudes and self-efficacy than those who identified as Christian or Muslim. This finding is partly consistent with that of previous studies on the association between attitudes and religion (e.g., [22]). In Ghana, while traditionalism provides a basis for understanding ID [9], this lens has also become the basis for the social exclusion and discrimination of persons with ID [12]. It was surprising that the participants who identified with the traditional religion were more efficacious and had more positive attitudes than others. There may be aspects of the traditional religion which might have influenced the participants; however, this particular inquiry was beyond the scope of this study. Furthermore, the understanding of Christians and Muslims about the onset and support for individuals with ID is unclear. Future studies could explore these areas to provide a better understanding of religion and ID in the Ghanaian context.

### Study limitations

As there are some study limitations, a generalisation of the findings would not be advisable. First, the participants were recruited from one public university as the opportunity to participate could not be extended to other students in other schools. However, the participants could represent healthcare students in other institutions. As a unitary country, where there is freedom of movement, individuals can move without restriction to other institutions to study. Therefore, the trends identified in this study could be similar in other institutions. Second, some religious groups were not captured in this study. Future studies could develop a comprehensive tool with the aim of capturing the various religious groups in Ghana. Nevertheless, the three main religious groups in Ghana were examined in this study. Thus, the results arguably reflect the perceptions of individuals based on religion. Third, it was beyond the scope of this study to verify whether the participants had taken courses in ID. Future studies could explore the contents of health curricular to assess the disability component, including its associated policies and programmes for rehabilitation. Despite these limitations, the study filled an important gap in the literature by including healthcare students’ perspectives regarding individuals with ID.

## Conclusion

The study reported here used Ajzen TPB [28] to assess the attitudes and self-efficacy of healthcare students towards individuals with ID in Ghana. With the negativity and subjugation of individuals with ID in Ghana [9, 10, 43], there is a need for support aimed at promoting their rehabilitation in society. For example, society has to understand the needs, uniqueness and strengths of individuals with ID before they can support their inclusion in productive activities. In achieving this goal, healthcare students have an important role to play in enabling individuals with ID to maximise their full potential. The attitudes and self-efficacy of healthcare students are fundamental to leading awareness creation and advocacy for persons with disabilities. However, the results of this study showed that the participants were ambivalent about both attitudes and self-efficacy. Also, there were interactions between attitudes and self-efficacy as well as differences between the participants on variables such as religion, having a relative with ID and the level and programme of study.

With limited formal support services for persons with ID, the role of healthcare students could be critical in advocacy and the creation of awareness about ID. Consequently, the results of this study could have implications for policymaking in Ghana. First, aside from training healthcare students about ID to enhance their understanding and change their attitudes, there should be efforts aimed at developing their self-efficacy so as to enable them to have more positive attitudes, be confident in their ability to support individuals with ID and create awareness among the larger populace. Second, health educators could consider exposing healthcare students to individuals with ID as part of their training. More structured field experiences aimed at increasing healthcare students’ contact and interaction with persons with ID is required. This would enable them to appreciate the uniqueness and needs of individuals with ID. The abstract training of healthcare students might not help in efforts towards improving their attitudes and self-efficacy to enable them to spearhead awareness. Third, it is important for health educators to wield the disability curricular around people’s culture and religion. This study has arguably provided corroborating support regarding the importance of religion towards understanding the attitudes and self-efficacy of healthcare students towards individuals with ID. Designing courses around religion and culture would help individuals understand areas considered pertinent to the understanding of ID in Ghana. There is a need to create a conducive environment for all persons, and considering some of these suggestions in curriculum development could help attempts at promoting the acceptance of persons with ID in productive activities in Ghana.

## Data Availability

The datasets generated and/or analysed during the current study are not publicly available due ethical restrictions but are available from the corresponding author on reasonable request.
